# Supplementary material for: CD112 Supports Lymphatic Migration of Human Dermal Dendritic Cells
Source: Cells. 2024 Feb 28;13(5):424. doi: 10.3390/cells13050424 (PMC10931060; doi:10.3390/cells13050424)
Supplement: Supplementary file 1 [file cells-13-00424-s001.zip › cells-2717982-supplementary.pdf]

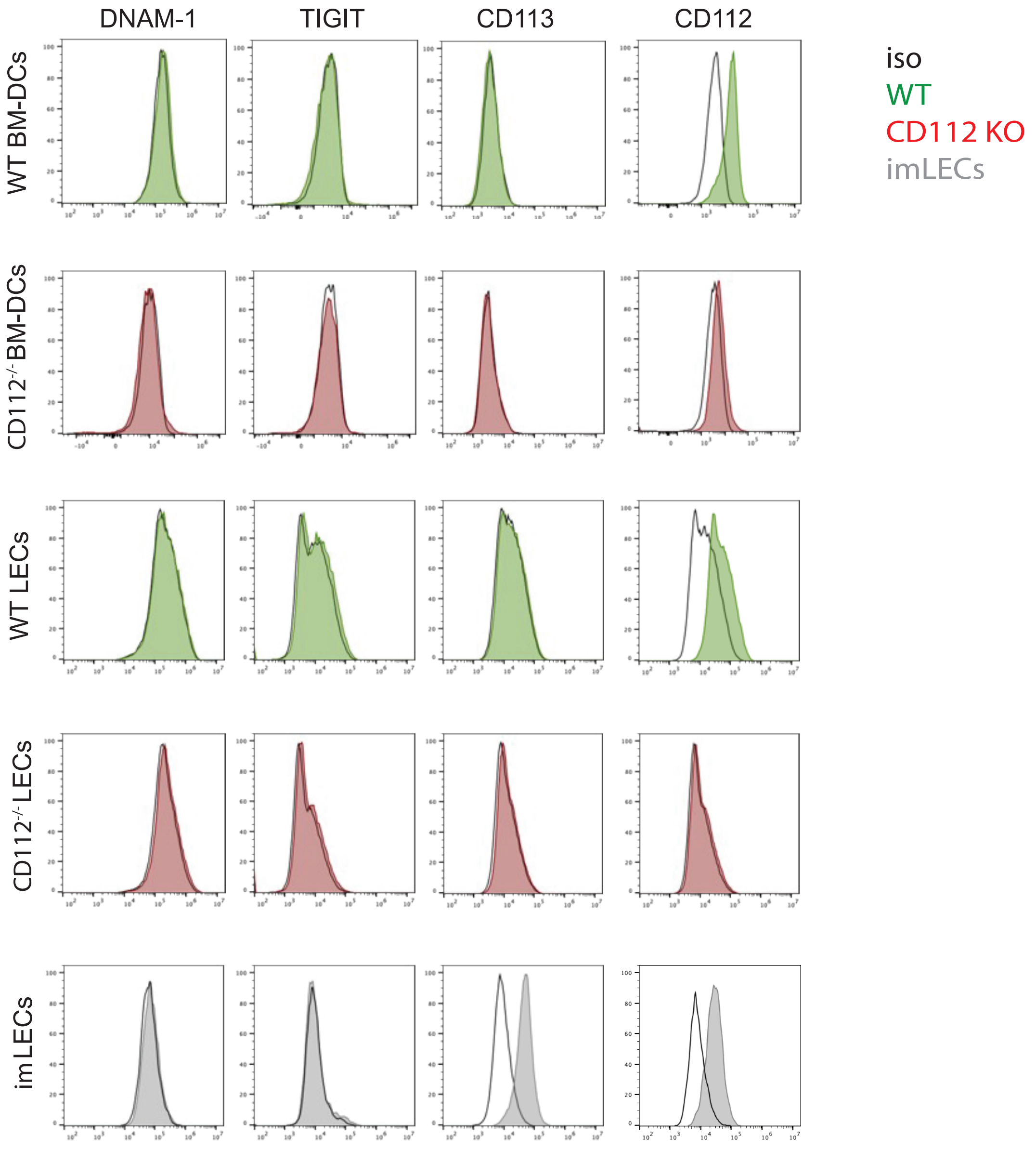

Suppl. Figure 1:**Expression of surface ligands on murine BM-DCs and murine LECs.** Flow cytometry analysis of known binding partners to CD112, in WT and CD112 KO BM-DCs and WT and CD112 KO LECs. In the case of TIGIT and DNAM-1, previously validated antibody clones were used [1]. In the case of CD113, the antibody was validated by staining conditionally immortalized LECs (imLECs). Representative histogram plots of >3 experiments are shown.

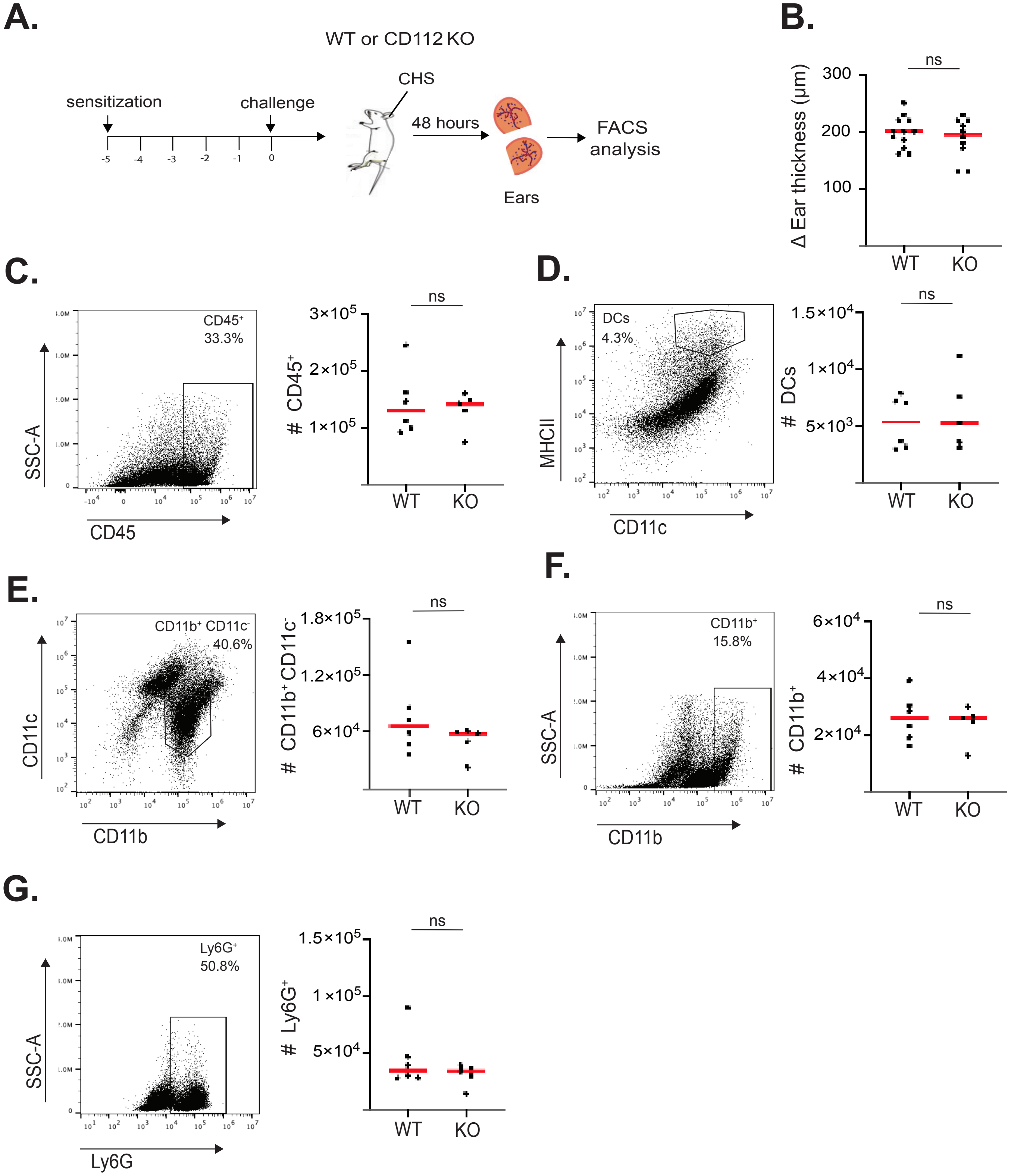

Suppl. Figure 2: No difference in the CHS response elicited in the ear skin of CD112 KO mice.
(A) Scheme of the experiment to analyze the composition of endogenous immune cells in ear skin upon CHS response. **(B)** Ear swelling response before and 48 hours after CHS response induced in the ears of WT or KO mice. Gating strategy and quantification of endogenous **(C)** CD45+ leukocytes, **(D)** CD11c+ MHCII+ DCs, **(E)** CD11b+ CD11c- cells, **(F)** CD11b+ macrophages, **(G)** Ly6G+ neutrophils in ear skin of WT or KO mice upon CHS response. Each dot represents an ear of mouse **(B)** or an individual mouse **(C,D,E,F,G)**.
Data obtained from one experiment with 5-6 mice per condition. The red line in graphs shows the median.
Mann-Whitney test was used and non-significant P values ($p>0.05$) have been shown as “ns”.

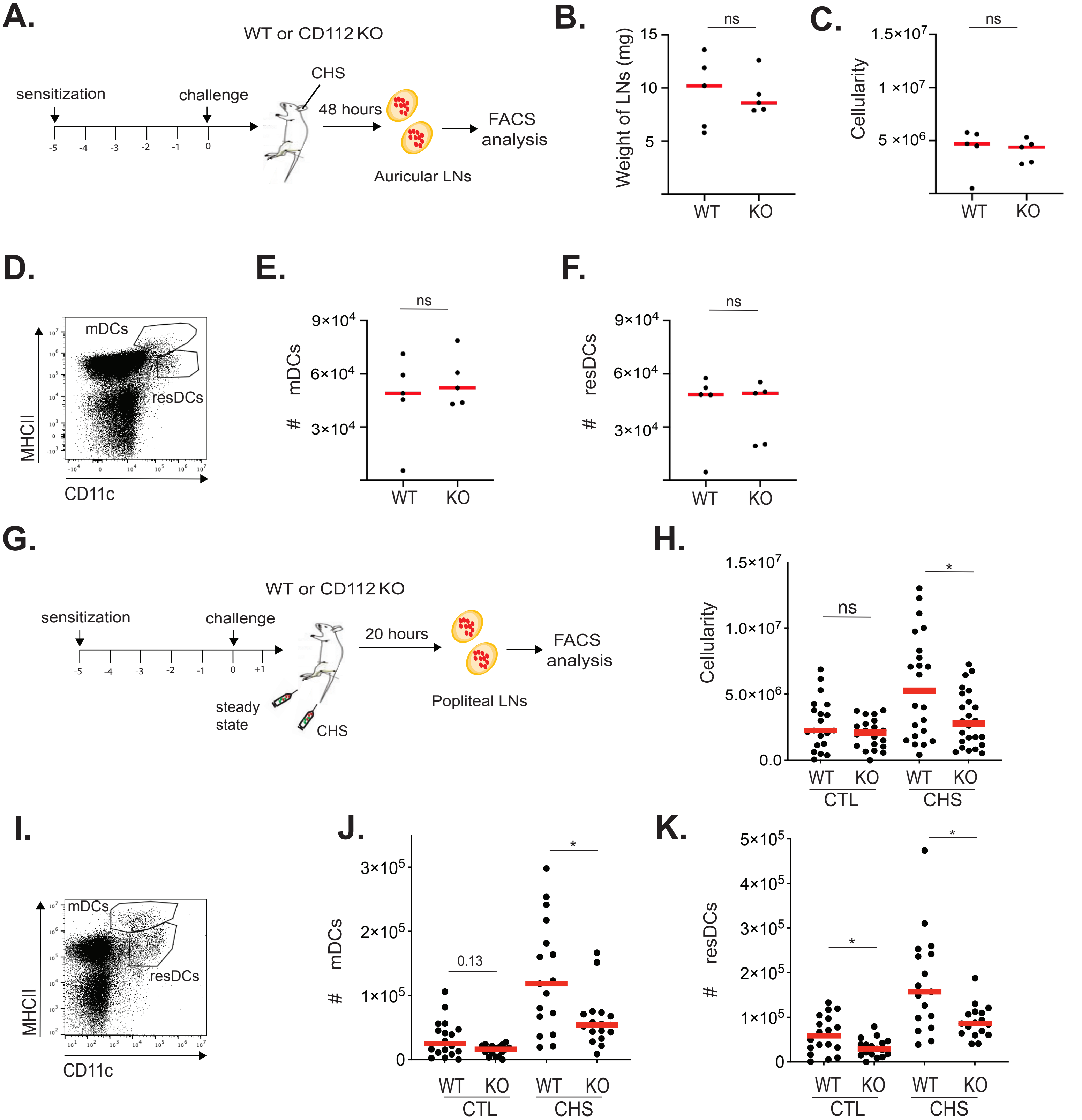

Suppl. Figure 3:Analysis of endogenous DC migration to dLNs upon induction of a CHS-response of WT and CD112 KO mice .(A) Scheme of the experiment to analyze the total number of DCs in auricular dLNs of WT or KO mice upon CHS response in ears. **(B)** Weight of auricular LNs and **(C)** cellularity after induction of CHS response in the ears of WT or KO mice. **(D)** Gating strategy to differentiate migratory (mDCs) and resident (resDCs) DCs in auricular LNs.Quantification of endogenous **(E)** mDCs and **(F)** resDCs in auricular LNs of WT or KO mice. **(G)** Scheme of the experiment to analyze the total number of DCs in popliteal dLNs of WT or KO mice after adoptive transfer of BM-DCs to the CHS-inflamed footpad. **(H)** Cellularity of popliteal LNs in steady-state and after induction of a CHS response in the footpad. **(I)** Gating strategy to differentiate migratory (mDCs) and resident (resDCs) DCs in popliteal LNs. Quantification of endogenous **(J)** mDCs and **(K)** resDCs in popliteal LNs drainingcontrol (CTL) or CHS-inflamed (CHS) footpads of WT or KO mice. Each dot represents a mouse (B,C,E,F,H,J,K). Data obtained from one experiment with 5 mice per condition (B-F), or three independent experiments with 4-8 mice per condition (H-K). The red line in graphs shows the median. Mann-Whitney test was used and P values indicate the following significances:*p<0.05;***p<0.001; ****p<0.0001.

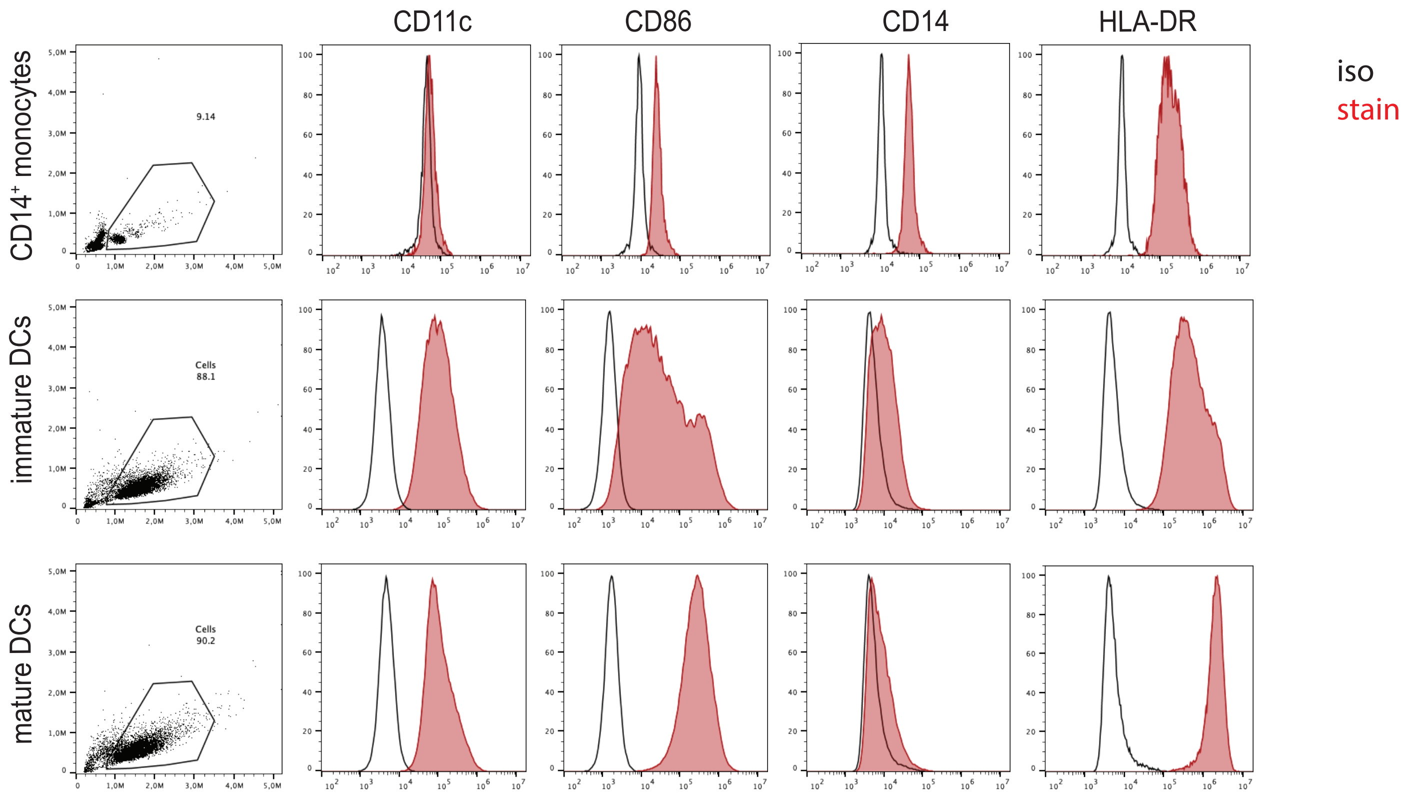

Suppl. Fig 4:Phenotypic analysis of human monocyte-derived dendritic cells (moDCs). Expression levels of CD11c, CD86, CD14 and HLA-DR in human moDCs, immature and LPS-matured DCs. Representative histogram plots of 3 experiments are shown.
